# Research on Texture Variation Mechanism of Ti-3Al-2.5V Titanium Alloy Tube During Cold-Rolling Process

**DOI:** 10.3390/ma19071282

**Published:** 2026-03-24

**Authors:** Huiyan Ge, Yumeng Luo, Boya Wang, Xiaoyun Song, Wenjun Ye, Yang Yu, Yanfeng Li, Songxiao Hui

**Affiliations:** 1 State Key Laboratory of Nonferrous Structural Materials, China GRINM Group Co., Ltd., Beijing 100088, China; ghy010520@163.com (H.G.); wangboya@grinm.com (B.W.);; 2 GRIMAT Engineering Institute Co., Ltd., Beijing 101407, China; 3 General Research Institute for Nonferrous Metals, Beijing 100088, China; 4 GRINM (Guangdong) Institute for Advanced Materials and Technology, Foshan 528051, China

**Keywords:** Ti-3Al-2.5V alloy, texture variation mechanism, ‘Q’ ratio

## Abstract

To investigate the mechanism of texture formation during the cold rolling of Ti-3Al-2.5V tubes for aerospace hydraulic systems, this study examines the microstructure at various locations of two deformation cones with ‘Q’ ratios of 1.055 and 1.300, respectively, in a single cold-rolling pass, revealing their continuous texture evolution. The results indicate that the cold-rolling texture primarily forms during the sinking section. A higher ‘Q’ ratio leads to a stronger tendency for the c-axis of grains to align parallel to the radial direction of the tube, resulting in enhanced radial texture intensity. Beyond influencing texture through dislocation slip, a higher ‘Q’ ratio also elevates the Schmid factor for {10$\overset{-}{1}$2} twinning. This twinning mechanism primarily forms the radial texture by altering the stress state. Consequently, this change not only facilitates twin activation but also modifies the rotation direction of grains during the twinning process. Compared to the cone with a ‘Q’ ratio of 1.055, the deformation cone with a ‘Q’ ratio of 1.300 contains a greater number of twins oriented along <0001>//RD, leading to a stronger radial texture in the tube.

## 1. Introduction

Ti-3Al-2.5V is an α-type titanium alloy primarily composed of a low-symmetry hexagonal close-packed (HCP) structure. Due to the extremely high precision requirements for aviation hydraulic tubes, they are typically mass-produced only through cold rolling. Ti-3Al-2.5V is a medium-to-high-strength titanium alloy whose mechanical properties satisfy the application requirements for aviation hydraulic pipeline systems. More significantly, in comparison with Ti-6Al-4V, Ti-3Al-2.5V possesses superior cold formability, which allows the production of aircraft hydraulic pipelines with stringent dimensional accuracy requirements by cold rolling. In addition to aviation applications, Ti-3Al-2.5V titanium alloy is also widely used in space shuttles, satellites, and other industrial fields [1,2,3].

At present, Ti-3Al-2.5V titanium alloy tubes are mainly produced by the two-roll periodic cold rolling tube method, which is called the Pilger cold-rolling method [4,5,6]. The formulation of processing techniques and the control of texture during its processing have always been the focus of research. The Compressive Strain Ratio (CSR) is a key performance parameter for tubular materials. It is defined as the ratio of circumferential plastic strain to radial plastic strain under tensile loading. This index characterizes the material’s resistance to wall thinning during bending processes and is known to be significantly affected by crystallographic texture [7].

In actual production, the ‘Q’ ratio, which is a parameter reflecting the relative variation between the radial and circumferential true strains, is commonly used as the main parameter to control the cold rolling process of tubes with an HCP structure [8]. Linga Mutry et al. [9] associated Q > 1 with wall-thinning-dominated deformation and Q < 1 with diameter-shrinkage-dominated deformation. Tenckhoff et al. [10,11] established that the cold-rolling ‘Q’ ratio dictates the deformation texture in zirconium alloy tubes: Q > 1 promotes a radial texture (c-axes parallel to radial direction), Q < 1 leads to a circumferential texture (c-axes parallel to circumferential direction), and Q = 1 results in a random basal pole distribution in the radial-circumferential direction.

Existing research systematically investigated the texture evolution of cold-rolled Ti-3Al-2.5V tubes and its correlation with deformation processes. Nie et al. [12] studied cold-rolled Ti-3Al-2.5V tubes and found that the tubes develop a {0002} texture along the radial direction, and a mixture of {10$\overset{-}{1}$0} and {11$\overset{-}{2}$0} textures along the axial direction. With increasing deformation, the orientations of both the radial {0002} poles and the axial {10$\overset{-}{1}$0} poles progressively rotate closer to the c-axis of the grains, resulting in a gradual decrease in their respective misorientation angles with the c-axis. Yang [13] indicated that the stress-strain state during the rolling process determines the plastic deformation mechanism of the tubes, which is the reason for the different types of textures formed. The primary purpose of controlling the ‘Q’ ratio is to regulate the strain magnitudes in the radial and circumferential directions during tube deformation, thereby influencing the plastic deformation mechanism and the resulting texture. The influence of ‘Q’ ratio on texture determines that ‘Q’ ratio affects the performance of Ti-3Al-2.5V tubes [14]. Chen et al. [15] proposed that a higher ‘Q’ ratio leads to more pronounced grain fibration and orientation. When the ‘Q’ ratio is controlled within the range of 1.54–2.46, the tubes exhibit favorable overall performance. Yang et al. [16] found that the ‘Q’ ratio has a significant influence on the tensile properties of the tubes. Overall, it is considered that a ‘Q’ ratio of 1.86–2.62 results in optimal cold-rolling effects for Ti-3Al-2.5V titanium alloy tubes.

The plastic deformation process in Ti-3Al-2.5V titanium alloy mainly relies on the activation of slip systems. Due to the scarcity of independent slip systems, twinning is often required to coordinate the continuity of strain during actual deformation [17,18,19]. Chen et al. [20] investigated the microstructure and texture evolution of Ti-3Al-2.5V titanium alloy tubes during cold rolling deformation. They found that deformation in the initial stage of tube rolling was dominated by prismatic slip. With an increase in rolling reduction, prismatic slip was successively replaced by basal slip and then pyramidal slip, resulting in a texture in the tubes primarily characterized by pyramidal components. Wei et al. [21] indicated that the strengthening of the radial texture is mainly attributed to the activation of tensile twinning {10$\overset{-}{1}$2} <10$\overset{-}{1}\overset{-}{1}$> during cold rolling. In multi-pass cold rolling processes, adopting a pass schedule design where the ‘Q’ ratio gradually increases with each successive pass is conducive to the formation of a stronger radial texture.

Based on previous research, the influence law of ‘Q’ ratio on texture was basically known, but the mechanism by which it realizes the change of cold-rolled texture is still unclear. Therefore, this study uses the intermediate product of the tube cold-rolling process, the deformed cone, to explore the change law of the tube texture during the cold-rolling process and reveal its change mechanism.

## 2. Experimental Materials and Methods

### 2.1. Experimental Materials

**Figure 1:**
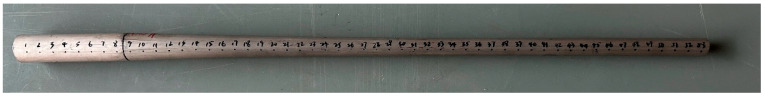
Physical Photograph of the Tube Deformation Cone.

**Figure 2:**
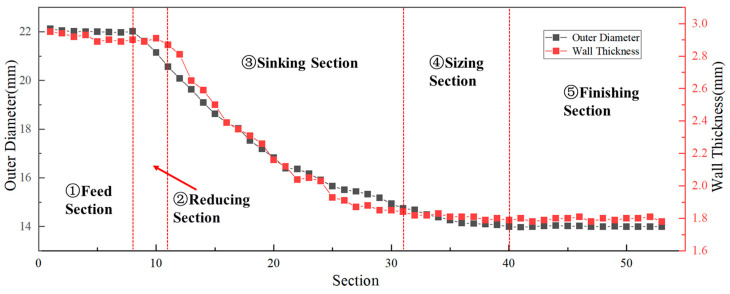
Tube Deformation Cone No.1: Dimensional Evolution.

**Figure 3:**
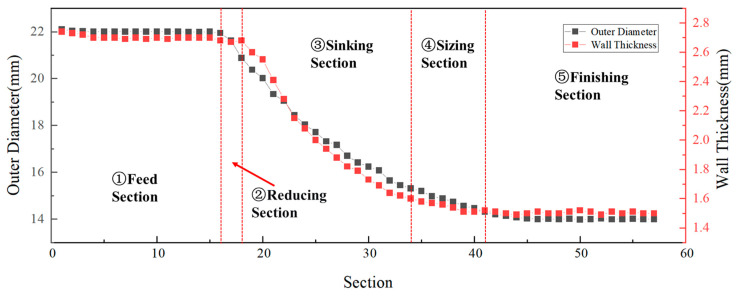
Tube Deformation Cone No.2: Dimensional Evolution.

Table 1 presents the chemical composition of the experimental workpieces, Ti-3Al-2.5V titanium alloy. The experiment workpieces used in this study were semi-finished tube cones obtained during the tube rolling process. Thus, the deformation cones encompass the entire process of the microstructural revolution during a single cold-rolling pass. Two cold rolling processes with distinct ‘Q’ ratios (1.055 and 1.300) were selected to prepare Ti-3Al-2.5V titanium alloy tubes. Both cones were manufactured using the Pilger cold rolling method. The design approach for their deformation spreading curves follows the sequence of diameter reduction first, followed by wall thickness reduction, and, finally, finishing. As a result, the dimensional variation patterns exhibit similarities. Using these processes, tubes were rolled from Φ22 × 2.9 mm to Φ14 × 1.8 mm and from Φ22 × 2.7 mm to Φ14 × 1.5 mm, with an error range of ±0.02 mm for each dimension, respectively. Figure 1 shows the tube deformation cone. The cone was divided with marks on its surface along the axial direction every 10 mm. The deformation tube was then sectioned axially, and a vernier caliper was used to measure the outer diameter and wall thickness at each mark, while the measurement error each time was ±0.02 mm. Each position was measured three times during the cone dimension measurements and the average value was adopted. The dimensional changes of the two tubes are illustrated in Figure 2 and Figure 3. The red dotted lines in the figure indicate the boundaries dividing the cone into different sections.

Following the continuous contour of the deformation cone, the forming process comprisesd five sequential stages: feed section, reducing section, sinking section, sizing section and finishing section. Since the texture changes of the deformation cone mainly occurred in the reducing section and sinking section and remained relatively stable in the sizing section and finishing section, only the reducing and sinking sections were selected for observation. Along each deformation cone, five specific sampling locations were defined: the initial reducing section, the final reducing section, the initial sinking section, the middle sinking section, and the final sinking section.

### 2.2. Experimental Methods

To accurately characterize the radial texture of the tubes, samples were prepared by the expansion method [13] and were then observed by a JEOL F7900 scanning electron microscope (JEOL Ltd., Akishima, Tokyo, Japan) equipped with an OXFORD backscattered electron detector (Oxford Instruments Nanotechnology Tools Limited, Abingdon, UK). The test plane was the radial plane of the middle layer of the tubes. AD, TD, and RD represent the axial, circumferential, and radial directions of the tubes respectively. The test parameters were set at a magnification of 500× and a step size of 0.5 μm. The relevant pole figures, Inverse Pole Figure (IPF) maps, and Kearns factors were exported by AZtecCrystal 2.1 and OIM 7.3 software to characterize the microstructure and the texture of the tubes. In the EBSD experiments, three different locations on the same cross-section were examined, and the results showed no significant variation. Therefore, the experimental data from a representative location are presented.

The Kearns factor, which is widely used to describe the proportion of basal {0001} planes in tubes with an HCP structure, such as Ti and Zr, was obtained according to the EBSD data [9,22]. Here, fr, fa, and ft represent the Kearns coefficients in the radial, axial, and tangential directions, respectively, and satisfy the relationship fr + fa + ft = 1.

## 3. Results and Discussion

### 3.1. The Microstructure

**Figure 4:**
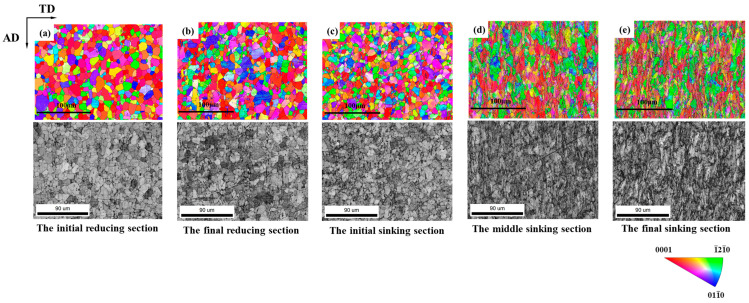
Microstructure Orientation Maps of Selected Sections in Deformation Cone No.1. (**a**) The Initial Reducing Section; (**b**) The Final Reducing Section; (**c**) The Initial Sinking Section; (**d**) The Middle Sinking Section; (**e**) The Final Sinking Section.

**Figure 5:**
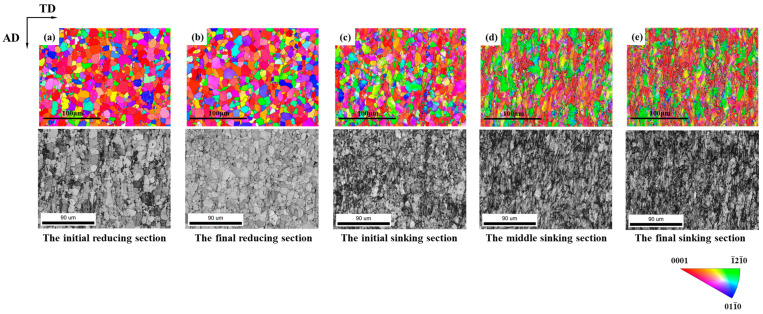
Microstructure Orientation Maps of Selected Sections in Deformation Cone No.2. (**a**) The Initial Reducing Section; (**b**) The Final Reducing Section; (**c**) The Initial Sinking Section; (**d**) The Middle Sinking Section; (**e**) The Final Sinking Section.

Figure 4 and Figure 5 show the IPF maps and the microstructure of the selected sections. It was observed that the grains showed no significant deformation during the reducing section, but elongated significantly along RD when entering the sinking section. For the deformation cone processed with a ‘Q’ ratio of 1.055, the preferred grain orientation gradually shifted from <0001>//RD towards <$\overset{-}{1}$2$\overset{-}{1}$0>//RD. In contrast, the preferred orientation in the deformation cone processed with a ‘Q’ ratio of 1.300 remained predominantly <0001>//RD throughout the entire process.

### 3.2. Texture Evolution

**Figure 6:**
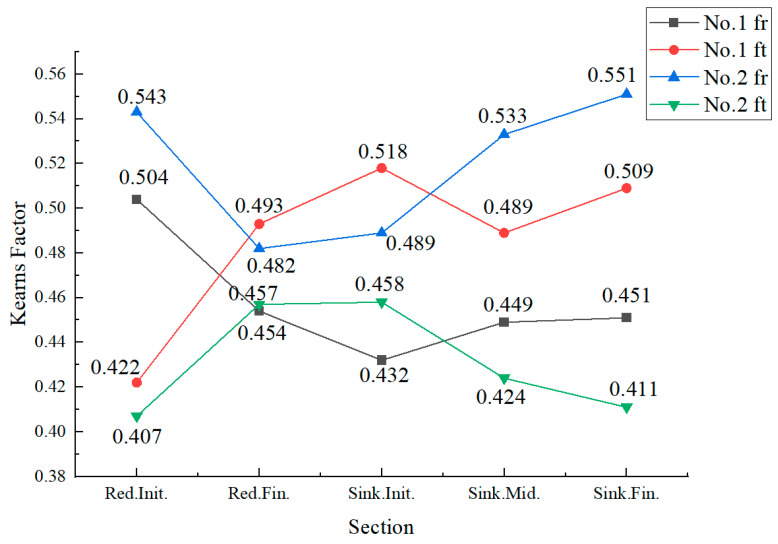
Variation Curve of Kearns Factor for the Deformation Cone.

The line chart of Kearns factors is shown in Figure 6. In Figure 6, the black and blue lines represent the radial Kearns coefficients (Kearns fr) of each cone, exhibiting a similar trend of initially decreasing and then increasing. The red and green lines represent the circumferential Kearns coefficients (Kearns ft) of each cone, showing a similar trend of initially increasing and then decreasing. The Figure 6 shows that during the deformation in both the reducing and sinking sections, the Kearns fr generally exhibited a trend of initial decrease followed by a subsequent increase. After rolling with a ‘Q’ ratio of 1.055, the texture of the cone transitioned from a pre-rolling radial texture to a circumferential texture. In contrast, the cone processed with a ‘Q’ ratio of 1.300 experienced a slight enhancement of its radial texture after rolling.

### 3.3. Mechanism of ‘Q’ Ratio Effect on Texture Evolution

#### 3.3.1. Dislocation Slip

During the deformation of the cone, grains underwent two typical types of changes. One was the overall reorientation within a grain, which is manifested in the IPF map as inhomogeneous coloration inside the grain. The other was the formation of lamellar grains with new orientations within the original grain. Both types of grain changes led to alterations in crystallographic orientation, thereby causing changes in the texture of the tube.

**Figure 7:**
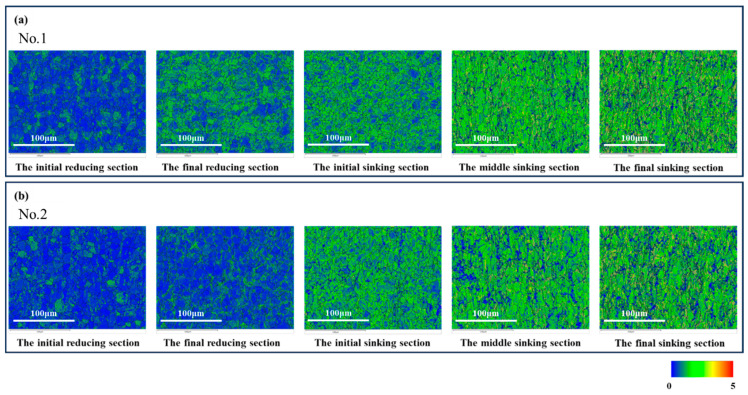
KAM Maps of Selected Sections. (**a**) Deformation Cone No.1; (**b**) Deformation Cone No.2.

Figure 7 shows the Kernel Average Misorientation (KAM) maps for the selected sections. It can be observed from the figure that the KAM values gradually increased as the rolling process proceeded. KAM reflects the degree of strain concentration, and a high KAM value typically corresponds to a region with high dislocation density. Therefore, it can be concluded that the dislocation density continuously rose during rolling, and regions with higher dislocation density exhibited relatively more active slip activity.

**Figure 8:**
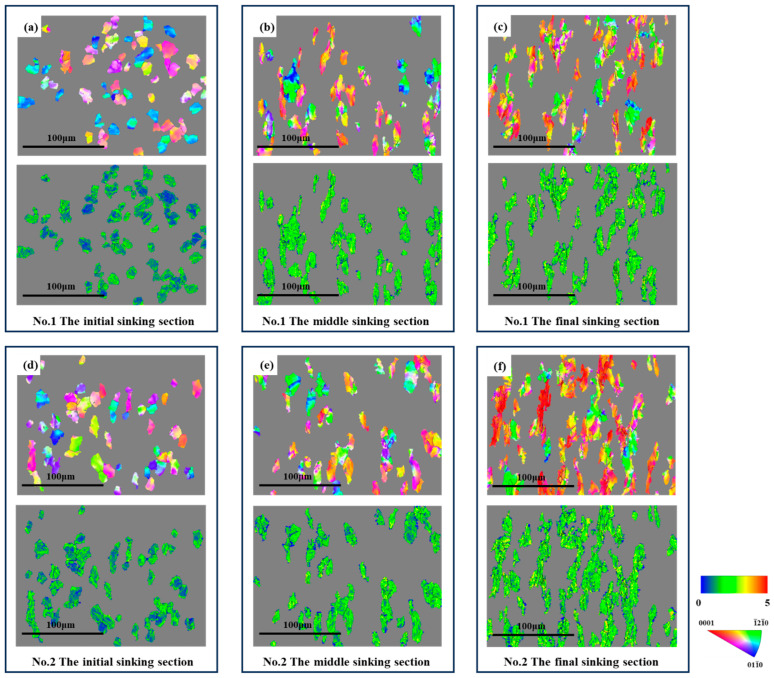
Rotated Grains and KAM Maps of the Microstructure of Various Sections of Deformation Cones. (**a**) Tube Deformation Cone No.1 Initial Sinking Section; (**b**) Tube Deformation Cone No.1 Middle Sinking Section; (**c**) Tube Deformation Cone No.1 Final Sinking Section; (**d**) Tube Deformation Cone No.2 Initial Sinking Section; (**e**) Tube Deformation Cone No.2 Middle Sinking Section; (**f**) Tube Deformation Cone No.2 Final Sinking Section.

Figure 8 highlights the grains with internal orientation inhomogeneity within the microstructure of the sinking section, where they underwent orientation changes due to dislocation movement during cold-rolling processes.

As observed in Figure 8 for deformation cone No.1, the initial region of the sinking section was primarily dominated by grains rotating between two specific orientations. These were the green <$\overset{-}{1}$2$\overset{-}{1}$0>//RD orientation and the blue <01$\overset{-}{1}$0>//RD orientation. With the advancement of cold rolling, the proportion of the two other types of rotating grains gradually increased. The first type encompassed yellow to orange grains, which rotated between the <$\overset{-}{1}$2$\overset{-}{1}$0>//RD and the <0001>//RD orientations. The second type included purple to pink grains, which rotated between the <01$\overset{-}{1}$0>//RD and the <0001>//RD orientations. By the end of the sinking section, yellow and pink grains became dominant.

In contrast, for deformation cone No.2, yellow and purple grains already accounted for nearly 50% in the initial region of the sinking section. With the continuation of cold rolling, the grains gradually rotated towards the <0001>//RD direction. By the end of the sinking section, most grains rotated to orange and pink orientations close to <0001>//RD.

**Figure 9:**
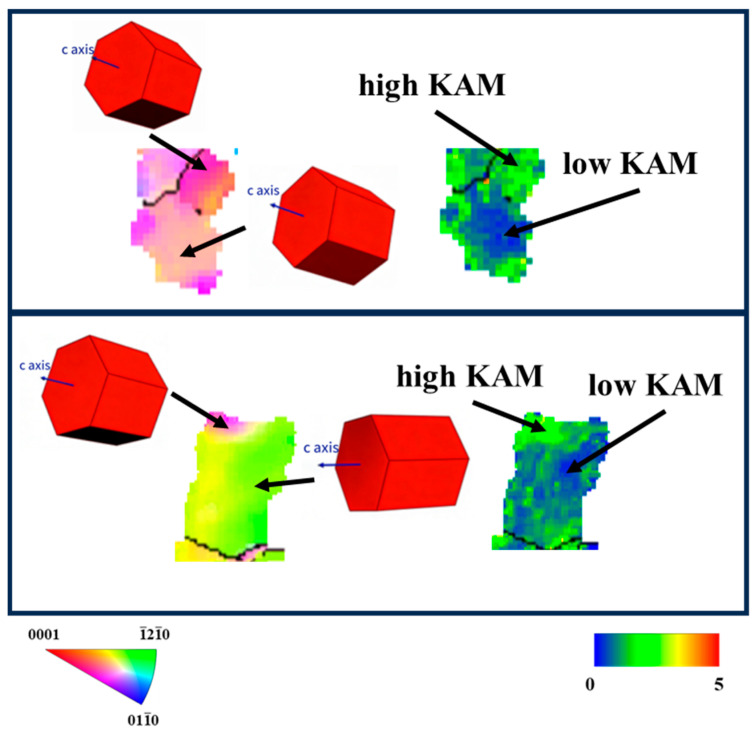
Schematic Diagram of KAM Distribution and Rotation Direction in a Typical Deformed Grain.

Combined with the KAM maps of deformed grains in Figure 8, it can be observed that grains with non-uniform IPF colors exhibited high KAM values. As the rolling process proceeded, the dislocation density continuously increased, and regions with higher dislocation density demonstrated relatively more active slip activity. Meanwhile, as shown in Figure 9, by the rotation direction and KAM values of the typical deformed grain, the activation of slip systems led to changes in grain orientation during the cold rolling of the tube.

A comparison of the two deformation cones reveals that a higher ‘Q’ ratio resulted in a more pronounced rotation of grains towards the <0001>//RD direction via activation of the slip system, ultimately resulting in a stronger radial texture in the final tube. Slip was a key factor responsible for the variation in the preferred orientation within the tube.

#### 3.3.2. Twinning

**Figure 10:**
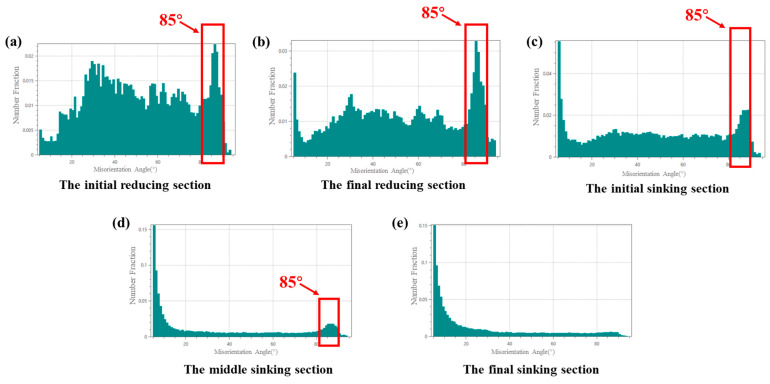
Statistics of Grain Boundary Misorientation Distribution for Deformation Cone No.1. (**a**) The Initial Reducing Section; (**b**) The Final Reducing Section; (**c**) The Initial Sinking Section; (**d**) The Middle Sinking Section; (**e**) The Final Sinking Section.

**Figure 11:**
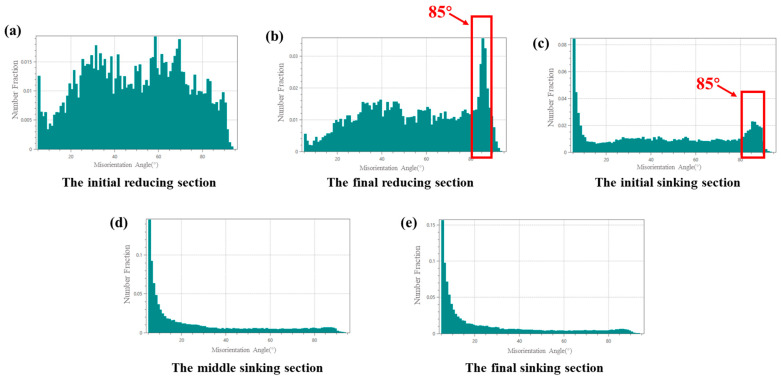
Statistics of Grain Boundary Misorientation Distribution for Deformation Cone No.2. (**a**) The Initial Reducing Section; (**b**) The Final Reducing Section; (**c**) The Initial Sinking Section; (**d**) The Middle Sinking Section; (**e**) The Final Sinking Section.

**Figure 12:**
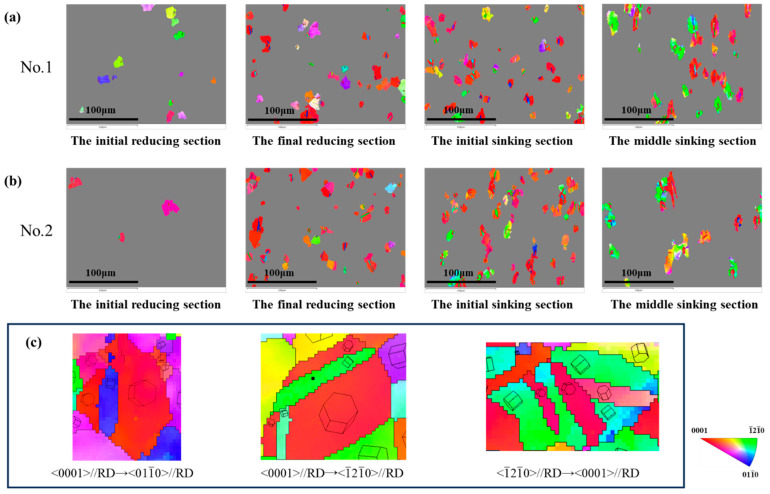
Highlighted Twin Microstructures in Selected Sections of Each Deformation Cone. (**a**) Highlighted Twin Microstructure in Deformation Cone No.1; (**b**) Highlighted Twin Microstructure in Deformation Cone No.2; (**c**) Orientations of Twinned Grains.

Figure 10 and Figure 11 present the microstructure orientation maps of the selected sections. As shown, a distinct peak appears at approximately 85°. All prominent 85° peaks in the figure have been highlighted with red boxes. Crystallographic characteristic angle analysis confirmed that these grains predominantly contained {10$\overset{-}{1}$2} tensile twins. In addition to grains with inhomogeneous coloration, a significant number of grains exhibiting straight grain boundaries and uniform color were observed across various stages of the deformation cone, as shown in Figure 12. The arrows in Figure 12c indicate the transformation of the grains.

The twin area fraction increased from the initial reducing to the sinking section from 8% to 12% for deformation cone No.1 and more markedly from 6% to 20% for cone No.2. Data from later sinking stages are omitted due to measurement challenges caused by severe deformation and grain refinement.

In the final reducing sections and initial sinking section, twinning predominantly occurred with a red-colored parent matrix oriented <0001>//RD and blue-colored twins oriented <01$\overset{-}{1}$0>//RD, as well as a red <0001>//RD matrix and green-colored twins oriented <$\overset{-}{1}$2$\overset{-}{1}$0>//RD. In the middle section of the sinking stage, the dominant twinning mode shifted to a green-colored matrix with <$\overset{-}{1}$2$\overset{-}{1}$0>//RD orientation and red-colored twins with <0001>//RD orientation.

By the final stage of the sinking section, twinning extended throughout the entire matrix, causing the grains to be completely occupied by the new twin orientations. Concurrently, severe grain twisting and fragmentation occurred during deformation, resulting in many twin boundaries deviating from the standard 85°, which made twin identification difficult. Under these conditions, the characteristic 85° peak was no longer present in the grain boundary misorientation distribution for this section. This transformation pattern aligns with the effects induced by dislocation activity. Therefore, both twinning and dislocation motion collectively contribute to the texture evolution during the cold-rolling process.

To understand this causal relationship, a mechanistic analysis of the phenomenon is required.

**Figure 13:**
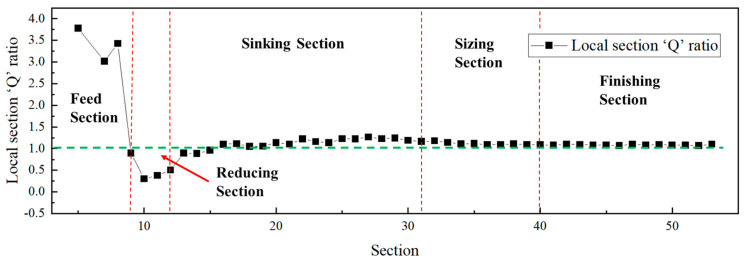
Variation Curve of Local Section ‘Q’ Ratio for Deformation Cone No.1.

**Figure 14:**
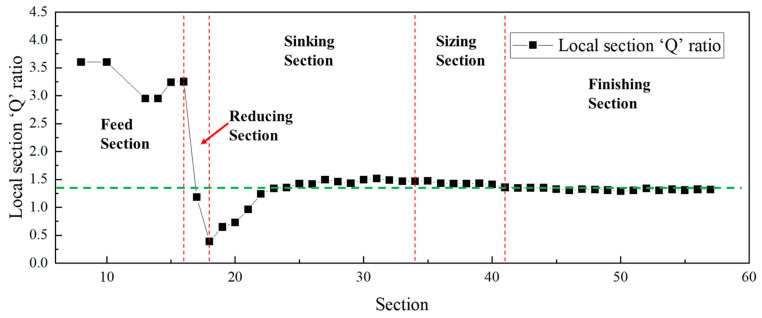
Variation Curve of Local Section ‘Q’ Ratio for Deformation Cone No.2.

The relationship between diameter reduction and wall thickness reduction in the ‘Q’ ratio essentially reflects the distribution of strain among different deformation directions. In the multi-pass Pilger cold rolling of Ti-3Al-2.5V titanium alloy tubes, the working cone can be modeled as a series of infinitesimally short annular deformation units. By analyzing the dimensional changes at various locations of the deformation cone, the strain variations in each direction at these positions can be determined. Furthermore, the local section ‘Q’ ratio can also be derived, as illustrated in Figure 13 and Figure 14. The red dotted lines in the figure represent the boundaries dividing the cone into different sections, while the green dashed line indicates the final ‘Q’ ratio of the deformation cone.

The curves indicate that the local section ‘Q’ ratio decreased significantly in the reducing section, reaching a minimum, and then increased during the sinking section until it attained the final rolling ‘Q’ ratio. This trend is consistent with the previously observed changes in the Kearns factors of the cones: the radial texture weakens in the reducing section and begins to strengthen from the sinking section onwards.

Figure 13 and Figure 14 indicate that the local sectional ‘Q’ ratio of the deformation cone decreased to below 1 in the reducing section, where deformation was predominantly diameter reduction. This occurred because the tube blank underwent a free diameter reduction process in this stage. During this phase, only the outer surface of the tube blank contacted the die groove, while the inner surface did not yet engage with the mandrel. Consequently, diameter reduction proceeded normally, while wall thinning deformed freely, leading to a progressively decreasing ‘Q’ ratio dominated by diameter reduction. In the sinking section, however, due to the deformation of the tube between the mandrel and zthe roll, the ‘Q’ ratio continuously increased, reaching its maximum by the end of this stage.

For cylindrical or annular tubular specimens, the radial, circumferential, and axial strains are typically represented by ε_r_, ε_θ_, and ε_z_, respectively, and they satisfy the condition $\varepsilon_{r}+\varepsilon_{\theta }+\varepsilon_{z}=0$. Within the cylindrical coordinate system (r, θ, z), shear deformation is relatively small and can be neglected. This allows the use of the tube rolling strain direction as a substitute for the stress direction in the analysis.

**Figure 15:**
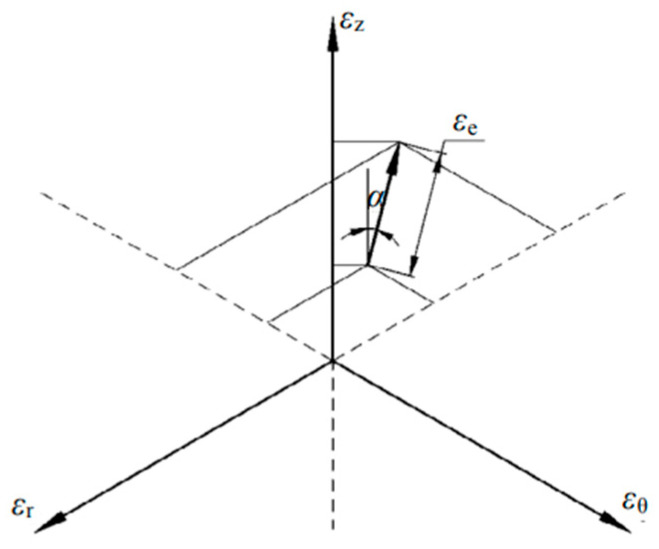
Diagram of Planar Three-axis Strain and Definition of the Plastic True Strain Vector [23].

For any infinitesimal segment of the conical deformation zone, a planar three-axis strain diagram can be employed to define the plastic true strain vector, as illustrated in Figure 15. The Figure 15 shows that the magnitude and direction of this true strain vector are directly related to the degree and mode of plastic deformation. Therefore, the effective strain ε_e_ and the strain ratio α are defined to quantify these characteristics, respectively [23].

The plastic true strain vector is given as follows:

(1) $$\varepsilon_{e}\;=\sqrt{\frac{2}{3}(\varepsilon_{r}^{2}+\varepsilon_{\theta }^{2}+\varepsilon_{z}^{2})}$$

(2) $$\alpha =\arctan\left(\frac{\varepsilon_{\theta }-\varepsilon_{r}}{\sqrt{3}\varepsilon_{z}}\right)$$

Twinning requires a certain critical resolved shear stress to be activated. The Schmid factor is used to characterize the ease of activating a specific twin system (defined by a twin plane and a twin direction) in a crystal under an applied stress state. The formula for calculating the Schmid factor is

(3) m=cosϕcosλ

where φ is the angle between the stress axis and the normal to the twin plane and λ is the angle between the stress axis and the twin direction.

**Figure 16:**
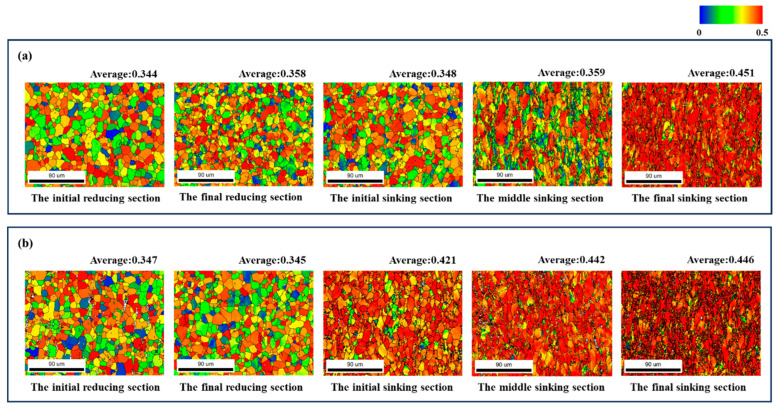
Distribution of Schmid Factors for {10$\overset{-}{1}$2} Tensile Twinning in Selected Sections of Deformation Cones. (**a**) Deformation Cone No.1; (**b**) Deformation Cone No.2.

From this formula, it is evident that the stress direction during tube rolling significantly influences the magnitude of the Schmid factor. By calculating the total strain and its direction from the three-dimensional strains and substituting them into Formula (3), the Schmid factors for the {10$\overset{-}{1}$2} tensile twinning in each section can be determined, as shown in Figure 16.

**Figure 17:**
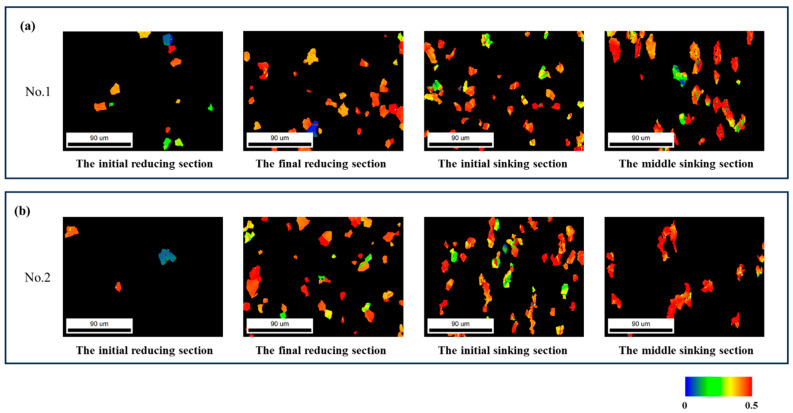
Distribution of Schmid Factors for {10$\overset{-}{1}$2} Tensile Twinning across Various Sections. (**a**) Deformation Cone No.1; (**b**) Deformation Cone No.2.

Comparing the local sectional ‘Q’ ratio with the distribution of {10$\overset{-}{1}$2} twinning Schmid factors in Figure 17 for the selected sections reveals that as the local sectional ‘Q’ ratio increased, the twinning Schmid factor also rose. Furthermore, the Schmid factors for deformation cone No.2 were generally higher than those for deformation cone No.1. A similar conclusion can also be drawn from merely observing the Schmid factor of twinning: for cross-sections at the same location under different local ‘Q’ ratios, the Schmid factor for twinning varied. A higher local sectional ‘Q’ ratio corresponded to a larger twinning Schmid factor, making twinning more likely to occur.

For deformation cone No.1, the area fraction of twins within the field of view increased from 8% in the initial reducing section to 12% in the initial sinking section. For deformation cone No.2, the twin area fraction rose more markedly, from 6% to 20% over the same stages. Statistical data on twin fraction from the middle to final sinking sections are omitted here due to measurement inaccuracies caused by severe deformation and grain refinement. These results demonstrate that an increase in the local ‘Q’ ratio promotes twinning, leading to a higher twinning Schmid factor and a greater twin area fraction.

Beyond the overall twin fraction, the local sectional ‘Q’ ratio also governs the orientation-dependent activation of twinning. Analysis combined with Figure 10 reveals a distinct pattern: When the local ‘Q’ ratio is less than 1, twinning occurs predominantly in red-colored grains, where the parent matrix orientation <0001>//RD transforms into twin orientations of <$\overset{-}{1}$2$\overset{-}{1}$0>//RD and <01$\overset{-}{1}$0>//RD. This process promotes a shift in the tube texture from a radial to a circumferential preference.

Conversely, when the local ‘Q’ ratio is greater than 1, twinning is activated more frequently in green-colored grains, involving a reorientation from a <$\overset{-}{1}$2$\overset{-}{1}$0>//RD matrix to a <0001>//RD twin. This facilitates a texture transition from circumferential to radial preference.

In summary, the texture evolution within the deformation cone is fundamentally governed by the local sectional ‘Q’ ratio. This parameter, which reflects the relative magnitudes of radial and circumferential true strains, primarily influences the twinning Schmid factor by dictating the strain distribution. The consequent alteration in twinning activation behavior is the key mechanism leading to the development of distinct texture types in the rolled tube.

## 4. Conclusions

(1) The texture evolution of cold-rolled Ti-3Al-2.5V tubes primarily completes during the reducing and sinking sections. Since the instantaneous ‘Q’ ratio shows a trend of first decreasing and then increasing in these two stages, the Kearns coefficient of the radial texture correspondingly decreases first and then increases. The final texture type of the tube is influenced by the magnitude of the total Q ratio, with tubes having Q > 1 exhibiting a stronger radial texture than those with Q ≈ 1. In practical production, it is recommended to use a cold rolling tube process with a ‘Q’ ratio greater than 1.3.
(2) During the rolling process, non-uniform dislocation slip occurs within some grains, leading to local orientation changes and influencing the texture type of the tube. In the cone with a higher ‘Q’ ratio, a larger proportion of grain areas undergoes dislocation-induced rotation toward the <0001>//RD orientation during rolling, thereby enhancing the radial texture of the tube.
(3) Through {10$\overset{-}{1}$2} twinning, a large number of grains originally oriented with <0001>//TD undergo a transition of their c-axis to align parallel to the RD, thereby forming a radial texture. The Q ratio alters the Schmid factor of {10$\overset{-}{1}$2} twinning by influencing the stress orientation. Compared to the condition of Q = 1, the Schmid factor of this twinning system is higher under Q = 1.3, which increases the probability of twinning and consequently enhances the radial texture of the tube.

## Figures and Tables

**Table 1:** Chemical composition of Ti-3Al-2.5V titanium alloy ingot. (wt.%).

Main Element, %			Other Element, %					
Ti	Al	V	Fe	C	N	H	O	Y
Bal.	3.02	2.50	0.148	0.005	0.003	0.001	0.084	<0.001

## Data Availability

The original contributions presented in this study are included in the article. Further inquiries can be directed to the corresponding authors.
